# Potential antiviral activities of chrysin against hepatitis B virus

**DOI:** 10.1186/s13099-023-00531-6

**Published:** 2023-03-09

**Authors:** Sajad Ahmad Bhat, Syed Kazim Hasan, Zahoor Ahmad Parray, Zaheenul Islam Siddiqui, Shabnam Ansari, Ayesha Anwer, Saniya Khan, Fatima Amir, Mahboubeh Mehmankhah, Asimul Islam, Zarrin Minuchehr, Syed Naqui Kazim

**Affiliations:** 1 Present Address: Centre for Interdisciplinary Research in Basic Sciences, Jamia Millia Islamia, New Delhi, 110025 India; 2 Department of Biotechnology, Jamia Millia Islamia, New Delhi, India; 3 Department of Biosciences, Jamia Millia Islamia, New Delhi, India; 4 National Institute of Genetic Engineering and Biotechnology, Tehran, Iran

**Keywords:** Hepatitis B virus, Chrysin, CccDNA, HMGB1, In silico

## Abstract

**Background:**

Interferon and nucleos(t)ide analogues are current therapeutic treatments for chronic Hepatitis B virus (HBV) infection with the limitations of a functional cure. Chrysin (5, 7-dihydroxyflavone) is a natural flavonoid, known for its antiviral and hepatoprotective activities. However, its anti-HBV activity is unexplored.

**Methods:**

In the present study, the anti-hepatitis B activity of chrysin was investigated using the in vitro experimental cell culture model, HepG2 cells. In silico studies were performed where chrysin and lamivudine (used here as a positive control) were docked with high mobility group box 1 protein (HMGB1). For the in vitro studies, wild type HBV genome construct (pHBV 1.3X) was transiently transfected in HepG2. In culture supernatant samples, HBV surface antigen (HBsAg) and Hepatitis B e antigen (HBeAg) were measured by enzyme-linked immunosorbent assay (ELISA). Secreted HBV DNA and intracellular covalently closed circular DNA (cccDNA) were measured by SYBR green real-time PCR. The 3D crystal structure of HMGB1 (1AAB) protein was developed and docked with the chrysin and lamivudine. In silico drug-likeness, Absorption, Distribution, Metabolism, Excretion and Toxicity (ADMET) properties of finest ligands were performed by using SwissADME and admetSAR web servers.

**Results:**

Data showed that chrysin significantly decreases HBeAg, HBsAg secretion, supernatant HBV DNA and cccDNA, in a dose dependent manner. The docking studies demonstrated HMGB1 as an important target for chrysin as compared to lamivudine. Chrysin revealed high binding affinity and formed a firm kissing complex with HMGB1 (∆G = − 5.7 kcal/mol), as compared to lamivudine (∆G = − 4.3 kcal/mol), which might be responsible for its antiviral activity.

**Conclusions:**

The outcome of our study establishes chrysin as a new antiviral against HBV infection. However, using chrysin to treat chronic HBV disease needs further endorsement and optimization by in vivo studies in animal models.

**Graphical Abstract:**

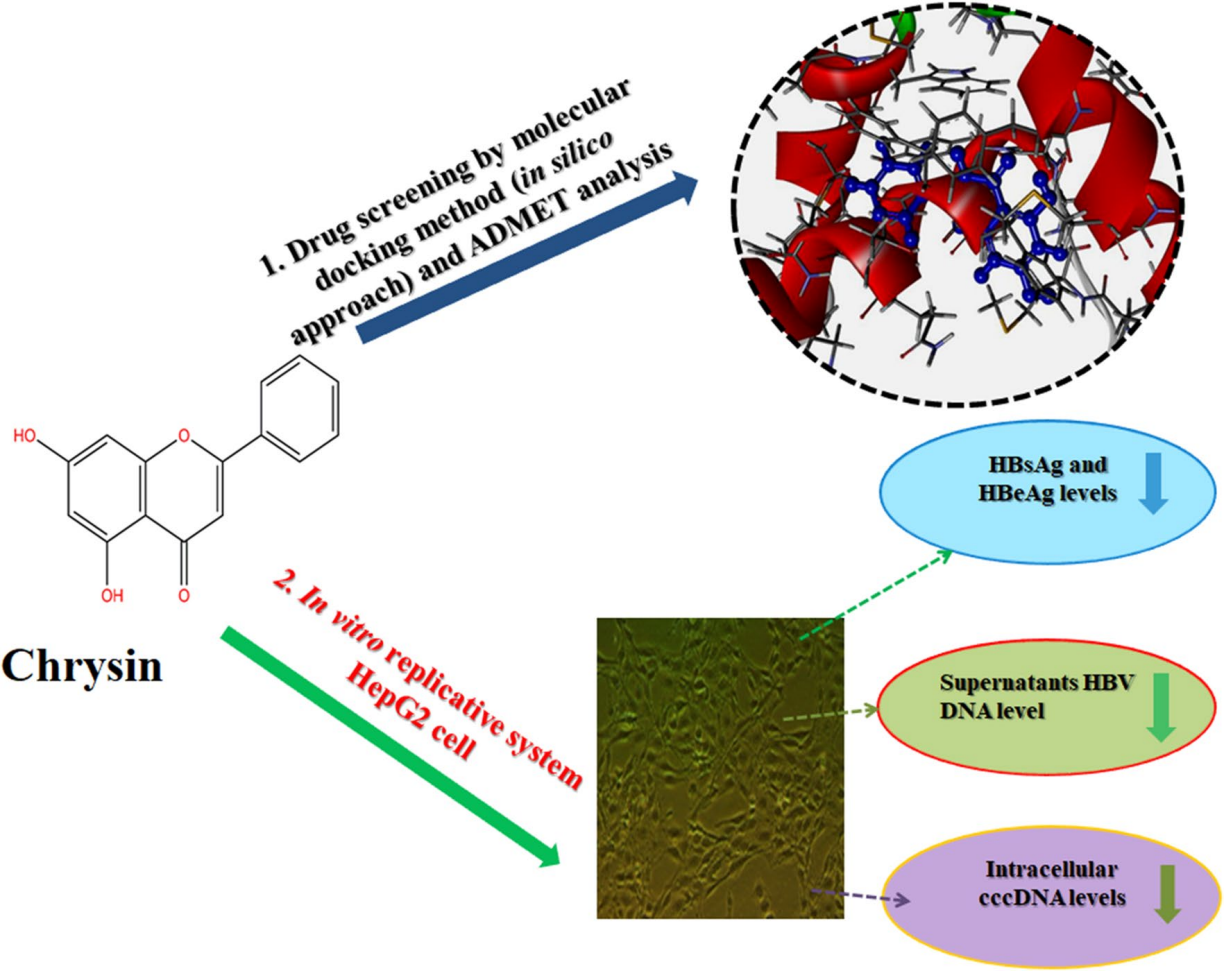

## Background

Chronic hepatitis B (CHB) disease is a predominant cause of liver cirrhosis and hepatocellular carcinoma (HCC) resulting in significant morbidity and mortality. This contributes to around one million deaths annually [1]. Despite the availability of an effective prophylactic genetically engineered vaccine, chronic hepatitis B continues to be a fundamental health challenge.

Hepatitis B virus (HBV), a member of family “*Hepadnaviridae*”, has a 3.2 kb partially double stranded, relaxed circular (rc) DNA genome [2]. The virion is made up of envelope protein and is surrounded by icosahedral protein capsid [3]. Upon entry into a hepatocyte, the HBV outer envelope is removed and the nucleocapsid is transported to the nucleus. The rcDNA genome is transformed into covalently closed circular DNA (cccDNA) in the nucleus. CccDNA, also known as minichromosome, is attached with chromatin and represents an essential element in HBV life cycle. CccDNA serves as template for the production of viral transcripts-pregenomic RNA (PgRNA), and PreC/C (3.5 kb), PreS1 (2.4 kb), S (2.1 kb), and X (0.7 kb) messenger ribonucleic acids (mRNAs) [4].

Currently, interferon (IFN-α), its pegylated form (PEG-IFN-α) and nucleoside analogues (NAs) are the anti-HBV candidates of choice and considered as gold standard for the treatment of chronic HBV infection [5, 6]. Though, none of them are effective. These effective candidates' viral specificity is a double-edged sword. Nucleoside analogues function by inhibiting viral HBV polymerase, thus impeding HBV replication. Its long-term administration causes genotype-dependent treatment response, dose dependent side effects, development of drug-resistant mutants, and a strong flare-up of HBV infection, besides the high cost of treatment [7]. Development of resistant HBV strains against most of the licensed antivirals is an emerging clinical problem [8, 9]. There are no available drugs that directly target the covalently closed circular DNA (cccDNA), an important replicative intermediate formed during replication cycle. The existing antivirals are ineffective in completely eradicating the nuclear pool of cccDNA [10]. Approximately 50% of patients on medication do not completely eliminate their viremia during the course of their treatment [11]. Screening of new drugs with effective anti-HBV potential, least or no toxicity, adverse side effects, drug resistance and novel mechanism of action is unquestionably important to combat chronic HBV infection. Consequently, it is important to produce safe, effective and promising anti-HBV candidates that impede viral replication and improve the clinical outcome of HBV affected patients.

The large repertoire of Traditional Chinese medicines (TCMs) possessing abundant natural herbs with antiviral and hepatoprotective properties are frequently used as additional medicines or as substitute to interferon-α and nucleoside analogues because they are less expensive and safer [12]. Traditional Chinese medicines, as a substitutive treatment have heralded a novel scope of therapeutic methods that lead us nearer to the hope to treat chronic HBV disease. In the past decade, ample clinical and experimental studies established various TCMs with tremendous antiviral potential against chronic HBV infection [13]. TCMs, being natural compounds, with diverse structures, offer ample opportunities for testing their anti-HBV potential with unique mechanisms of action [14].

Chrysin, also called 5, 7-dihydroxyflavone, has been used as TCMs for a long time. It is a ubiquitously occurring natural flavonoid found in honey, propolis, and a variety of plant extracts [15]. Antioxidant [16, 17], anti-allergic [18] anti-inflammatory [19], anti-fibrotic [20], anti-cancer [21, 22], and hepatoprotective activities have been demonstrated for chrysin. Chrysin grabbed our attention because of its known antiviral activities [23–25], though no published data is available on the anti-HBV activities of chrysin.

Chrysin has been shown to have antiviral activities, although the specific underlying anti-influenza mechanism and its anti-influenza effectiveness in vivo are still mostly unknown. Chrysin's role in blocking cell cycle and apoptosis in several cell lines exposed to two strains of the H1N1 influenza A virus (IAV) and its anti-IAV activity in vivo were examined. They demonstrated that chrysin significantly inhibited IAV replication via a mechanism independent of viral protein interaction and activation of the innate antiviral immune system. Chrysin, notably, can prevent IAV-induced cell cycle arrest in the G0/G1 phase by down-regulating the expression of P53 and P21 and favouring the activation of Cyclin D1/CDK4 and Cyclin E1/CDK2. Chrysin also significantly reduced caspase-9 and caspase-3 activation, altered the balance of Bax/Bcl-xl, and blocked the IAV-triggered mitochondrial apoptotic pathway. Chrysin favoured inhibiting IAV replication in the upper respiratory tract, suggesting that it may be a promising therapeutic drug for preventing the respiratory viruses transmission [26].

Chrysin demonstrated exceptional affinity for SARS-CoV, MERS-CoV, and SARS-CoV 2 Mpro. Furthermore, Chrysin blocked ACE-2 from interacting with the S protein of SARS-CoV-2 [27]. In line with these broad spectrum antiviral activities of chrysin, we reported similar kinds of results in the present study.

In this study we evaluated the effect of chrysin against HBV antigenic secretion in transfected liver cells with pHBV 1.3X wild type recombinant construct containing more than full length genome. We have noticed that chrysin efficiently inhibited secretory proteins in dose-dependent manner. The formation of both HBV covalently closed circular DNA (cccDNA) and  extracellular HBV DNA were reduced by chrysin treatment to a higher extent, representing that chrysin compound targeted replicative intermediate of DNA synthesis. Lack of appropriate animal models makes it herculean to understand the action mechanism of chrysin which is a drawback of present study [28].

In the present study the natural compound chrysin as well as lamivudine standard was docked with High-mobility group box 1 protein (HMGB1). HMGB1 is a nuclear element, signalling biological molecule, important DNA, RNA binding protein, associated with chromatin which serves as a mediator in both acute and chronic inflammation [29]. It is a multifunctional alarmin that plays a predominant role in many biological functions like transcription, DNA repairs and cell development [30]. Furthermore, it has been documented that it boosts the concentration of many proinflammatory cytokines known to play a vital role in triggering liver inflammation in chronic HBV infection, such as tumour necrosis factor (TNF)-α which is linked with chronic HBV disease [31]. HMGB1 a “leaderless cytokine” was found to be an important proinflammatory molecule indicates that many valuable anti-inflammatory drugs could use their action mechanism by impeding its proinflammatory effects and reduce the inflammation rate. Our outcomes of the present in vitro and in silico studies demonstrated novel anti-HBV properties of chrysin.

## Materials and methods

### Compound

Chrysin, a natural compound of cell culture grade was obtained from Sigma-Aldrich Company (St. Louis, MO, USA). Dimethylsulphoxide (Sigma-Aldrich, USA, 100%) was used for dissolving chrysin and 5 mM stock concentration was prepared. Serial dilutions of drug were performed with the Dulbecco’s Modified Eagle's medium (DMEM) cell culture media to get variable concentrations used for in vitro studies. For subsequent experiments, the drug stock was stored in opaque container at 4 °C. The natural compound as well as the other reagents was of molecular biology grade.

### Maintenance of cell line

Human hepatoma cell line HepG2 was obtained from National Centre for Cell Sciences (NCCS), Pune, India. Cells were cultured under standard cell culture conditions at 37 °C in a controlled humidified atmosphere with 5% CO_2_ supply and 100% humidity in CO_2_ incubator (NuAire NU-5830, USA). Culture was done in 25cm^2^T- flask (Nunc, Roskilde, Denmark) in Dulbecco’s Modified Eagle's medium (DMEM) enriched with nutrients such as glucose, sodium bicarbonate, sodium pyruvate,4-(2-hydroxyethyl)-1-piperazineethanesulfonic acid (HEPES) buffer supplemented with 10% (v/v) heat inactivated Fetal Bovine Serum (FBS), 1% (v/v) Penicillin–Streptomycin 10,000 U/mL solution (all from Invitrogen, San Diego, CA, USA**).** The cells were then collected from the flask using 0.25% trypsin (Gibco-BRL, Grand Island, NY, USA) and 1 mmol/L EDTA, and then revived for subsequent investigations. In the drug treatment experiments, cells with passage numbers of 2 to 15 were employed.

### Cell toxicity

Prior to the study of antiviral activity against HBV, the cytotoxic effects of the experimental compound were assessed. 3-(4, 5- dimethyl-2-thiazolyl)-2, 5-diphenyl-2-H-tetrazolium bromide) (MTT) (Sigma, St. Louis, MO, USA) assay was employed to assess the viability and cytotoxicity, as reported earlier [32]. Briefly, 2 × 10^4^ cells were seeded per well in 96 well plates (CoStar, Corning Inc., NY, USA) and incubated for 6–48 h. The cells were treated with variable concentrations (0, 2.5, 10 and 15 µM) of chrysin and incubated for 72 h. After treatment, MTT solution was prepared by dissolving in PBS (pH 7.4) with final concentration of 0.5 mg/mL. MTT reagent of 100 µL was added in all wells including control. Plate was again kept in culture incubator for 2 to 4 h. Dimethyl sulfoxide (100 µL) was added in each well to dissolve the formazan crystals. After 20 min of incubation, the dissolved dye was quantified spectrophotometrically by taking absorbance at OD 450 nm by an ELSIA plate reader (Bio-Rad, Hercules, CA, USA). The percent cell viability was calculated as per the formula:

$$\% \,{\text{Viability}}\,{ = }\,{{{\text{mean}}\,{\text{OD}}_{{{\text{sample}}}} } \mathord{\left/ {\vphantom {{{\text{mean}}\,{\text{OD}}_{{{\text{sample}}}} } {{\text{mean}}\,{\text{OD}}_{{{\text{control}}}} }}} \right. \kern-0pt} {{\text{mean}}\,{\text{OD}}_{{{\text{control}}}} }} \times 100$$

Chrysin showed its effect on cell viability. IC_50_ was calculated from the GraphPad Prism version 8.0 software (San Diego, CA. USA). The experiments were performed in triplicates in order to confirm reproducibility. The safer doses derived from this method were employed in subsequent studies.

### Transfection with pHBV 1.3X plasmid

The pHBV 1.3X construct harbouring more than full length HBV genome was a kind gift from Dr. Joseph Kock, (Heidelberg, Germany) and Dr. Shiv Kumar Sarin (ILBS, Delhi, India). Briefly, HepG2 cells (2 × 10^5^ cells/well) were seeded into 6-well plate and incubated for 24 h. For transfection, 1 μg of (pHBV 1.3X) wild type construct combined with 3μL of Lipofectamine 2000 (Invitrogen, Carlsbad, CA, USA) was used per well (Costar, Corning Inc., NY, USA), according to the Lipofectamine 2000 manufacturer instructions.

### Drug treatment to transfected HepG2 cells

The non-cytotoxic doses derived from MTT assay were used in subsequent studies. HepG2 cells were treated with safe doses (2.5, 5, 10 and 15 µM) of chrysin and incubated for 72 h. After 72 h post-transfection, culture supernatants were collected for the assessment of HBsAg, HBeAg, and extracellular HBV DNA. The cells were harvested with trypsin digestion, washed three times in phosphate buffered saline (PBS, pH 7.3). The amount of viral DNA in the cellular extract was measured.

### Assessment of HBsAg and HBeAg in cell culture supernatant

To measure the HBV secretory proteins from culture supernatants of drug treated cells, the enzyme-linked immunosorbent assay (ELISA) was performed using commercially available ELISA kits-Hepalisa (J Mitra &Co, Delhi, India) and DIA. PRO, MI, (Italy) for HBsAg and HBeAg, respectively. According to the manufacturers’ instructions we performed the analysis of HBsAg and HBeAg in the culture supernatant. In order to confirm the reproducibility of independent experiment, assays were performed in triplicate. The data, presented here as % inhibition of HBsAg and HBeAg, were calculated by the following formula:

$${\text{\% inhibition }} = \, {{\left( {{\text{OD}}_{{{\text{control}}}} {-}{\text{ OD}}_{{{\text{Sample}}}} } \right)} \mathord{\left/ {\vphantom {{\left( {{\text{OD}}_{{{\text{control}}}} {-}{\text{ OD}}_{{{\text{Sample}}}} } \right)} {{\text{OD}}_{{{\text{control}}}} \, \times {\text{ 100\% }}}}} \right. \kern-0pt} {{\text{OD}}_{{{\text{control}}}} \, \times {\text{ 100\% }}}}$$

### Assessment of HBV DNA in the supernatant

After 72 h of post-treatment, we observed the effect of compound on the quantity of the extracellular HBV DNA. The culture supernatant was collected by centrifugation at 1200 rpm for 10 min at 4 °C and HBV DNA was isolated and quantified by qPCR (Roche Applied Science, Penzberg, Upper Bavaria, Germany) employing the QIAmp DNA Mini Kit (Qiagen, Hilden, Germany) following the manufacturer’s instructions. The oligonucleotide sequence of forward primer was 5′-CCG TCT GTG CCT TCT CAT CTG-3′, the sequence of reverse primer was 5′-AGT CCA AGA GTA CTC TTA TAG AAG ACC TT-3′, and the sequence of Taqman probe was FAM-CCG TGT GCA CTT CGC TTC ACC TCT GC. The PCR program performed included an initial denaturation at 94 °C for 2 min trailed by 40 amplification cycles with each of the two subsequent steps: 95 °C for 5 s and 60 °C for 30 s. Plasmid containing more than the full-length insert of the HBV genome was used to form a standard curve. The standard curve exhibited a satisfying linear range when around 10^2^–10^7^ copies of plasmid DNA were used as template. The inhibitory effects of chrysin on HBV DNA were calculated by the following formula:

$${\text{\% inhibition = }}{{\left( {{\text{Copy number of control}}{-}{\text{ Copy number of sample}}} \right)} \mathord{\left/ {\vphantom {{\left( {{\text{Copy number of control}}{-}{\text{ Copy number of sample}}} \right)} {{\text{Copy number of control }} \times {\text{100\% }}}}} \right. \kern-0pt} {{\text{Copy number of control }} \times {\text{100\% }}}}$$

### Purification and quantification of intracellular HBV cccDNA

The effect of chrysin on the level of intracellular cccDNA was observed 72 h post-treatment. For this, cccDNA was isolated from the cell pellet containing 1.0 × 10^6^ cells using mini plasmid extraction Kit (QIAGEN Inc., Chatsworth, CA, USA) following the manufacturer’s instructions. The isolated plasmid was further treated with plasmid safe ATP-dependent DNase (PSAD, Epicentre Technologies, Madison, WI, USA), for 2 h at 37 °C, to eliminate HBV relaxed circular DNA (rcDNA), residual single-stranded viral DNA and cellular chromosomal DNA. This ATP-dependent DNase degrades linear single-stranded and double-stranded DNA, but acts moderately on closed circular double-stranded DNA. The real-time fluorescent quantitative PCR was performed with gene specific primers and Taqman TAMRA fluorescence hybridization probe to detect cccDNA. The sequence of forward primer was 5′-ACT CTT GGA CTC TCA GCA ATG-3′, sequence of reverse primer was 5′-CTT TAT AAG GGT CGA TGT CCA-3′ and sequence of Taqman probe was FAM-CTT TTT CAC CTC TGC CTA ATC ATC TCT TGT TCA- TAMRA. Because of the structural dissimilarities between cccDNA and rcDNA, only cccDNA will be amplified with the designed primers and probe set. PCR conditions were: denaturation for 2 min at 95 °C, followed by 38 cycles of denaturation at 94 °C for 15 s, 58 °C for 30 s, 72 °C for 30 s. The relative quantification of cccDNA was calculated using 2^−∆∆Ct^ method as previously described [33].

### Chemical structures and molecular docking studies

In the present study in silico approaches were also used  to trace the active compounds against Hepatitis B virus. It is a convenient drug screening method, where the candidate drugs can be virtually examined at low cost in a short duration. This involves computational simulation of target-ligand interaction and regulates the perfect alignment of binding of one molecule to the second molecule to generate a stable complex. This method, called docking, is used to find the activity of binding of a tiny molecule (in this case, a drug candidate- chrysin, and a positive control lamivudine) to their protein target (in this case HMGB1) by using the scoring functions.

This screening method plays a pivotal role in the functional designing of drug candidates.

### Target preparation

HMGB1 (PDB ID: 1AAB) is a signalling protein present in the nucleus. Its crystal structure was downloaded from protein data bank (http://www.pdb.org/). We removed the crystallographic water molecules or ligands, in order to produce a free receptor, and to enhance the entropy of the target. The missing polar hydrogen atoms were incorporated, and the energy level of target was minimized while using Swiss_PDB viewer tool. With the help of this server, we speculated the 3D structure of HMGB1 protein. Autogrid 4 module, a bioinformatics tool used to map the protein’s 3D structure, covered all the amino acid residues of the protein. The grid three dimensions X, Y and Z were fixed to be 50, 70 and 58 Å (receptor axis coordinates), and 0.405 Å as grid space size for lamivudine-HMGB1; and 52, 83 and 58 Å (receptor axis coordinates), and 0.435Å as grid space size for chrysin–HMGB1.

### Ligand preparation

**Fig. 1:**
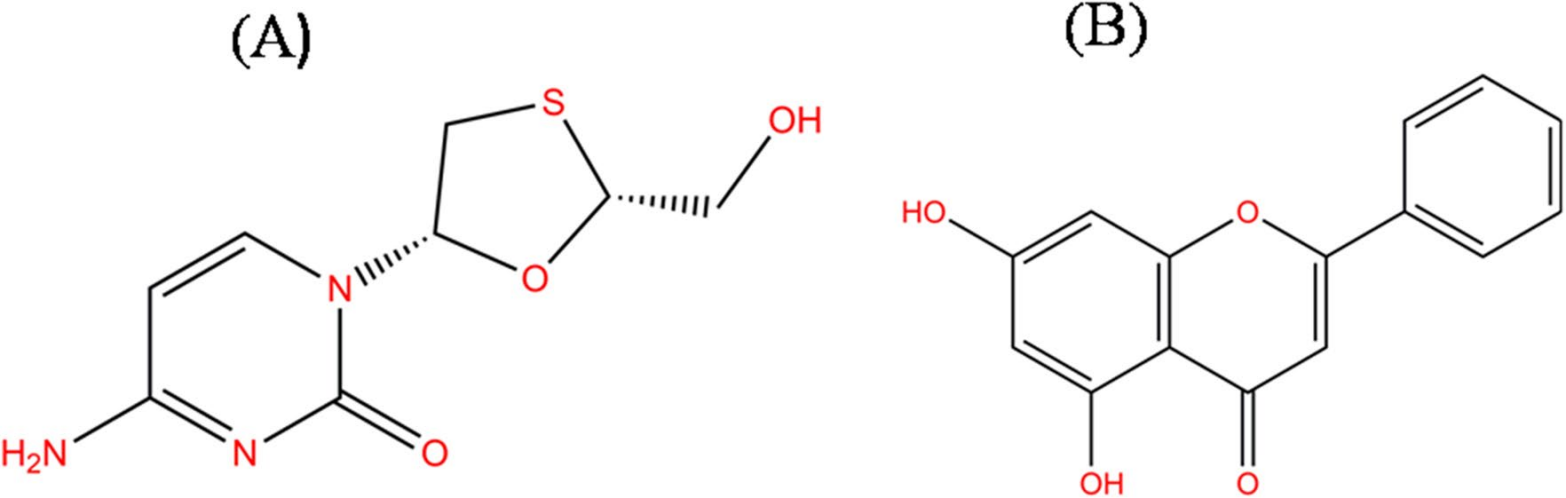
**A** and **B** represents optimized two-dimensional molecular geometries of anti-HBV compounds lamivudine (nucleoside analogue used as a reference drug in molecular docking analysis only) and chrysin respectively

The molecular structure of lamivudine (positive control) and chrysin was drawn by ChemDraw12 (PerkinElmer Informatics, Waltham, MA, USA) as shown in Fig. 1A and B, respectively. The molecular structure of compounds was converted into 3D form, and geometries were optimized in ChemBio3DUltra12 (PerkinElmer Informatics, Waltham, MA, USA). For docking studies, the tested compound chrysin and positive control lamivudine was saved in PDB format. Molecular docking was performed by using Auto dock 4.2 in order to achieve better insights into the binding mechanism of chrysin and lamivudine with HMGB1. Docking guidelines were followed in this docking simulation. To achieve molecular docking, Lamarckian Genetic Algorithm (LGA) was used to define the best potential structures of the ligands that directly interacted with the target protein. Here the ligand was allowed free to explore and interact with the protein's active site in the best possible or threshold energy configuration. Ideal docked configurations were archived and studied for further interaction between receptor-ligand, employing BOVIA Discovery Studio 4.0 to generate 2D interaction plot [34]. Docking was eventually visualized by Pymol [35]. Lamivudine, a nucleoside analogue approved by FDA for the treatment of chronic hepatitis B virus infection, was also docked with the same protein. In the molecular docking analysis, lamivudine was used as a positive control. For protein–ligand interaction, the binding constant (Kb) was calculated using equation **(**∆G = − RTlnKb (*R* = universal gas constant, 1.987 kcal/mol/; *T* = temperature, 298 K) [36].

### ADMET examination

The drug-likeness analysis was performed by admetSAR and SwissADME to confirm any cytotoxicity produced by ligands in humans. Several pharmacokinetics properties such as absorption, distribution, metabolism, excretion and toxicity (ADMET) of the tested compound chrysin and the positive control lamivudine were measured with the web tools admetSAR [37] and SwissADME [38]. The physicochemical properties were also studied with the help of these servers.

### Statistical analysis

All the results were presented as mean ± standard deviations (SD) of triplicate experiments standardized to control. In the present study, a standard statistical test such as one-way analysis of variance (ANOVA) was done, and data were analyzed to evaluate where precise variances were existing between the samples. Moreover, to define statistical significance Dennett’s t-test was used. GraphPad Prism software version 8.0 was used to calculate the mean and standard deviation, and draw graphs for the results of ELISA, MTT, HBsAg, HBeAg and viral DNA assays. MTT assay was expressed in terms of percentage viability, percentage cytotoxicity and IC_50_ value, p < 0.05 was considered statistically significant (*p < 0.05, **p < 0.01, ***p < 0.001).

## Results

### Effects of chrysin on viability of HepG2 cells

**Fig. 2:**
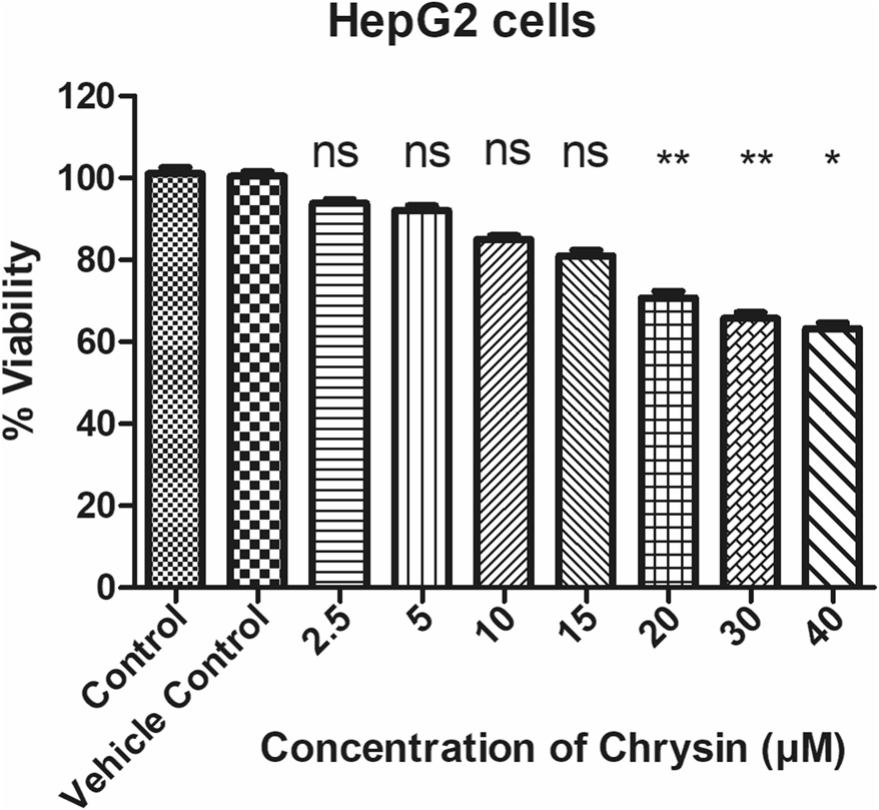
Represents cytotoxicity of chrysin measured by MTT assay. Cells seeded in 96-well plate and were incubated overnight. Cells were treated with different concentrations of chrysin. Untreated wells act as a control. In an ELISA reader, the absorbance of the MTT formazan was determined at 450 nm. The percentage of absorbance (OD) of treated cells to untreated cells was measured as % viability. Data presents mean ± standard deviations (SD) of three independent experiments carried out in triplicate. ^∗^p < 0.05, **p < 0.01, compared with control

Any potential harmful effect of chrysin on HepG2 cells was investigated using MTT assay prior to starting research on anti-HBV activity. In HepG2 cells, any effect of experimental drug (chrysin) on HBV may get exaggerated by cytotoxicity of the drug itself. Thus, assessing cytotoxicity of chrysin on viability of HepG2 cells is important. For further cell culture assays, the viabilities of HepG2, treated with variable concentrations of chrysin (2.5, 5, 10, 15, 20, 30, 40 µM) were studied after 72 h incubation by MTT assay. Figure 2 shows viability analysis of chrysin which demonstrate that HepG2 viability was almost unaffected up to 15 µM but when the concentration exceeded 15 µM, the cells lost its viability gradually. This also confirms that chrysin did not exhibit any significant effect on cells up to 15 µM, and 20, 30 and 40 µM doses were lethal to cells because these concentrations produced cytotoxicity and therefore were excluded for subsequent experiments. The results also confirmed that test compound maintained viability of cells at concentrations of 2.5–15 µM. Meanwhile, the calculated IC_50_ of chrysin on HepG2 was 22.5 µM. In a dose dependent fashion, the cells lost viability. Percentage viability of cells at concentrations 2.5, 5, 10, 15, 20, 30 and 40 µM of chrysin was 92.8%, 92.8%, 85%, 83%, 75.2%, 68% and 65.6% respectively. However, 2.5, 5, 10 and 15 µM were the favourable doses for subsequent studies.

### Effects of chrysin on HBV virions

**Fig. 3:**
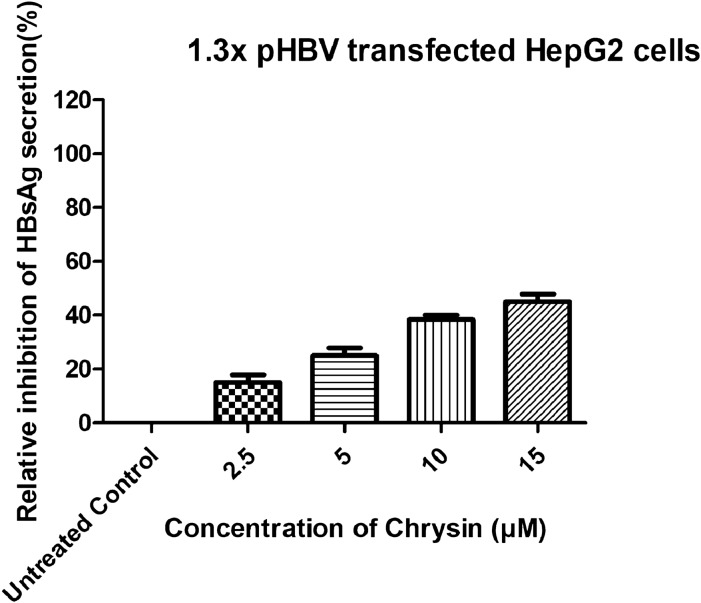
Inhibitory effects of chrysin, in a dose dependent manner, on the levels of HBsAg in HepG2 cells transfected with 1.3X pHBV (replication competent) plasmid. Commercial ELISA kit was used for the detection of HBsAg in the culture supernatants after 72 h incubation. The data are presented as mean ± S.D from three independent experiments

**Fig. 4:**
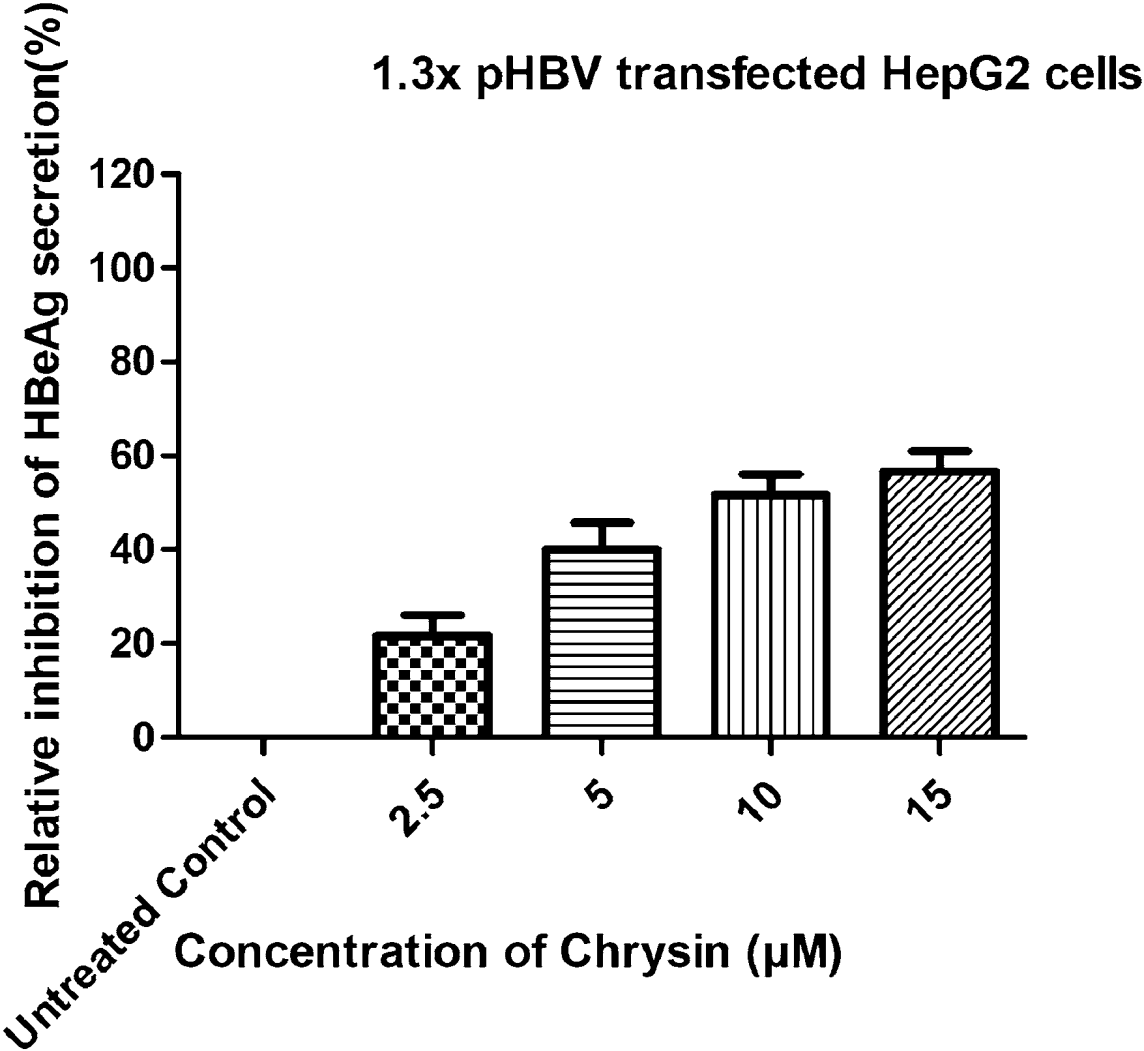
Inhibitory effects of chrysin, in a dose dependent manner, on the levels of HBeAg in HepG2 cells transfected with 1.3X pHBV (replication competent) plasmid. Commercial ELISA kits were used for the detection of HBeAg in the culture supernatants after 72 h incubation. The data represents mean ± S.D from three independent experiments

To assess the anti-HBV effects of chrysin on the in vitro cell culture system, transformed human hepatoma cell line HepG2 was used that has been transiently transfected with 1.3X pHBV construct. HepG2 was selected because it has been widely used for studying viral life cycle, especially with respect to formation of viral replicative intermediates and cccDNA [39]. Before finding the potential doses that affect HBV replication, it is important to use variable concentrations that are not overtly cytotoxic. Any damage to cell or its functions would interfere virus replications. Therefore, safe doses obtained by MTT assay were used for demonstrating antiviral effects. Effect of chrysin on the expression of viral antigens was studied. HepG2 cells were treated with variable concentrations (2.5, 5, 10 and 15 µM) of chrysin. HBV antigens (HBsAg and HBeAg) secretion was demonstrated in the culture supernatants or cell lysates using ELISA. Treatment of HepG2 cells with chrysin for 72 h leads to a decline in extracellular HBsAg and HBeAg production and the effects were dose-dependent (Figs. 3 and 4) (p < 0.05 or p < 0.01), the inhibitory effect evidently seemed for extracellular HBsAg or HBeAg. The extracellular HBV DNA level was also altered with variable chrysin doses. It was believed that the inhibition of HBsAg and HBeAg might be associated with direct interaction between chrysin and HBV antigen. As understood from Figs. 3 and 4, chrysin could encourage considerable inhibition of HBsAg and HBeAg. The inhibitory potential of chrysin on the secretion of HBeAg was more pronounced than that on the secretion of HBsAg. Chrysin inhibited the secretion of HBsAg and HBeAg by 18%, 25%, 38%, 45% and 25%, 40%, 49%, 58%, respectively (p < 0.05) at a concentration of (2.5, 5, 10 and 15 µM). These results showed that the tested compound could statistically down-regulate secretory proteins levels.

### Effects of chrysin on the replication of extracellular HBV DNA

**Fig. 5:**
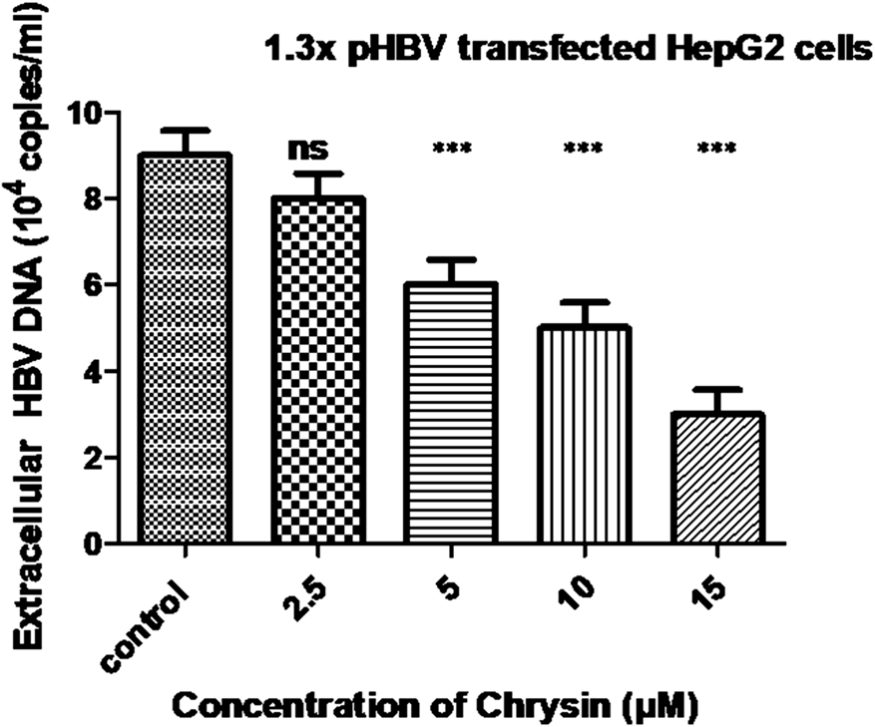
Inhibitory effect of chrysin on the secretion of extracellular HBV DNA in HepG2 cells transfected with 1.3X pHBV in a dose dependent manner. Cells were seeded into 24 well plates and were subjected to the tested compound chrysin and subsequently incubated for 72 h. Samples of HepG2 culture supernatant was collected and the HBV DNA quantified by real-time PCR. Untreated cells acted as control. Each experiment was done in triplicate. The data represents mean ± SD from three independent experiments (ns is non-significant, ***p < 0.001 compared with control)

The quantity of supernatant HBV DNA reveals the HBV replicating potential. Furthermore, to evaluate the efficacy of chrysin against HBV in HepG2 cells, the influence of drug on HBV DNA level in culture supernatant was assessed by safe doses with real-time quantitative PCR. Usually, the levels of secretory proteins (HBeAg, HBsAg) and DNA will be impeded when the HBV replication is inhibited. Treatment of the cells with variable concentrations of chrysin (2.5, 5, 10 and 15 µM) led to a numerically substantial decrease in the level of extracellular HBV DNA as compared with the untreated control. After chrysin treatment for 72 h in a dose dependent fashion, the HBV DNA content in the culture supernatant decreases. The percentage inhibitions of extracellular HBV DNA were 21%, 28.5%, 34.4% and 61% at 2.5, 5, 10 and 15 µM respectively (Fig. 5). However, our mechanistic studies showed that chrysin affects HBV DNA replication, and the percentage inhibition of chrysin on HBsAg and HBeAg reveals that the HBeAg and HBsAg secretion was certainly obstructed in the presence of chrysin. It was observed that chrysin considerably decreased the HBV DNA replication compared to the untreated control (p < 0.01). Our results showed that chrysin had anti-HBV potential in hepatocyte cultures in vitro.

### Inhibitory effects of chrysin on the genesis of intracellular HBV cccDNA pool 

The activity of chrysin on the level of intracellular HBV cccDNA was also studied. After HBV virion entry into hepatocytes, cccDNA is the first replicative intermediate produced, which demonstrates the beginning of intracellular HBV replication and effective development of HBV infection. After cessation of antiviral therapy, cccDNA stubbornness in hepatocytes nuclei is believed to be largely accountable for the recurrence of chronic HBV disease. Thus, the effect on nuclear cccDNA is an essential parameter when assessing an anti-HBV candidate, as an indicator of long-term antiviral response to treatment.

**Fig. 6:**
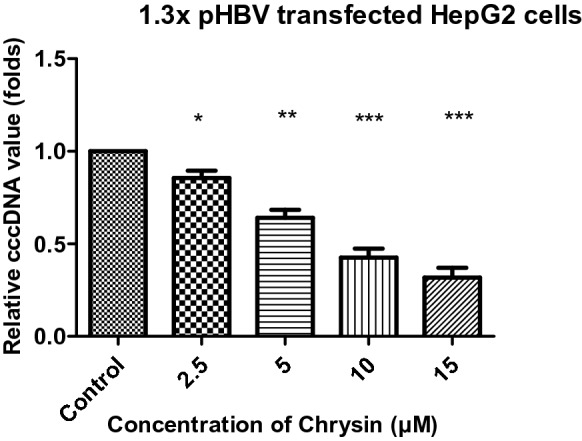
Inhibitory effects of chrysin on cccDNA in a dose dependent manner after 72 h of treatment. Cells in untreated wells serve as control. Data are presented as mean ± SD of three independent experiments. ^∗^p < 0.05, **p < 0.01, ***p < 0.001 and ****p < 0.0001 compared with control

To understand the mechanism of action of chrysin, we tried to examine the effect of chrysin on HBV replication. The level of HBV transcription template (cccDNA) was estimated by using quantitative real-time PCR. We observed that the tested compound chrysin significantly inhibited HBV cccDNA in a dose dependent manner. Enhancing the doses of chrysin caused the inhibition gradually in the formation of intracellular cccDNA as demonstrated in Fig. 6. After treatment with variable concentration of chrysin (2.5, 5, 10 and 15 µM) for 72 h, the intracellular cccDNA reservoir were down regulated by 14.4%, 35.9%, 57.4% and 68.1% respectively as compared with the untreated control. The results suggest that chrysin has a robust inhibitory potential on the genesis of cccDNA pool in a cell culture system. Taken all data together, chrysin might play an unprecedented role in protecting against chronic HBV infection.

### Molecular docking study

#### Interaction between lamivudine and HMGB1

**Fig. 7:**
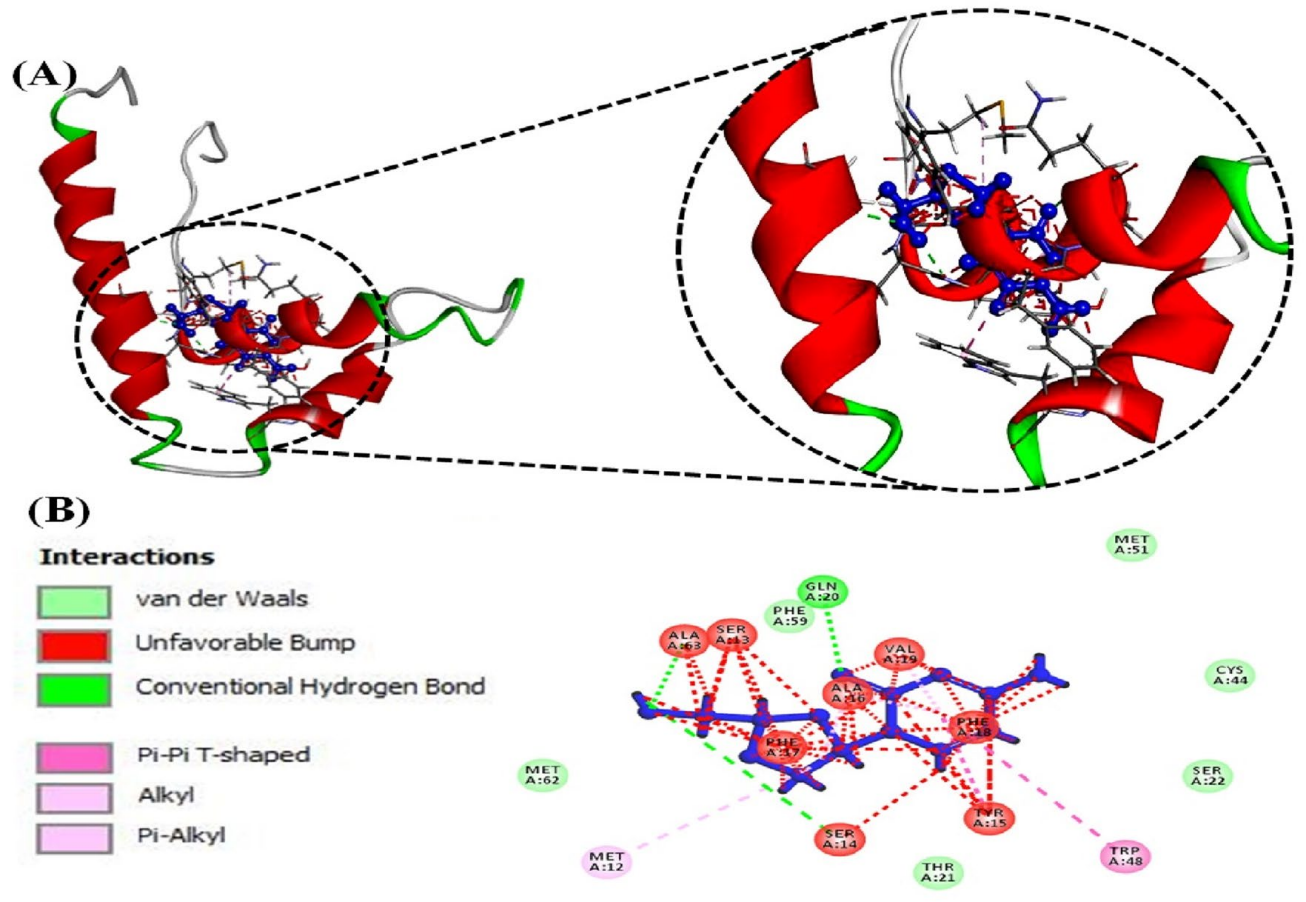
**A** Ball and socket cartoon model (blue) of the ligand (lamivudine) positive control interacting with the protein (HMGB1), **B** 2-D representation of various types of interactions (showed in color codes) of the ligand (lamivudine) in ball and socket model (blue) with specific amino acid residues of the protein

Compound lamivudine was docked with HMGB1 protein (PDB ID: 1AAB). The binding energy of lamivudine − 4.3 kcal/mol shows strong affinity with target protein that reflects a binding affinity of 1.0 × 10^3^ per mole (Table 1). Figure 7A provides a cartoon model of the protein as red-green ball and stick pattern. Lamivudine (blue) interacts with various amino acid residues of the protein receptor. Figure 7B provides 2D-plot representation of ligand (lamivudine) interaction with various amino acids of the protein, in which the main residues include GLN20, MET12, SER13, SER14, PHE17, PHE59, ALA16, VAL19, TYR15, and TRP48 via various types of interactions (conventional H-bonding, Pi-Pi T-shaped, Alkyl, Pi-alkyl, van der Waals forces, unfavourable accepter-accepter, unfavourable donor-donor bonds etc.). Table 1 provides residues of the protein that interact with the ligand, various types of interaction, and bond distances between the protein and the drug.

**Table 1:** Binding sites and various types of interactions occurring between drugs lamivudine (positive control) and chrysin with HMGB1 protein

Compounds	Amino acid interacted	Type of interactions	Bond distance (Å)	^b^∆*G* (kcal mol^−1^)	^c^*K*_b_ (/mole)
^a^Lamivudine (Nucleoside analogue)	*GLN20, *MET12, *SER13, *SER14, PHE17, PHE59, *ALA16, *VAL19, TYR15, and *TRP48	Conventional Hydrogen bonding, Van der Waals interactions, Pi-Pi T-shaped, Alkyl, Pi-alkyl, unfavourable acceptor-acceptor and unfavourable donor-donor bonds	3.54 4.91 4.15 4.26 5.01 4.82 4.26 3.25	− 4.3	1.0 × 10^3^
Chrysin	*MET12, ASP 66, MET 62, ALA63, PHE59, GLU60, HIS33, *GLN20, ARG23, CYS44, *VAL19, PHE17, PHE18, *SER14, SER22, PHE 40, *TRP48, *ALA16, and *SER13	Conventional H-bonding, van der Waals forces, Pi-Pi T-shaped, Alkyl, Pi-alkyl, unfavourable acceptor-acceptor and unfavourable donor-donor bonds etc.	3.77 4.14 3.12 5.28	− 5.7	14 × 10^3^

^a^Standard/positive control

^b^Binding energy or Gibbs free energy

^c^Binding affinity

^*^Indicates some common interacted amino acids

#### Interaction between chrysin and HMGB1

**Fig. 8:**
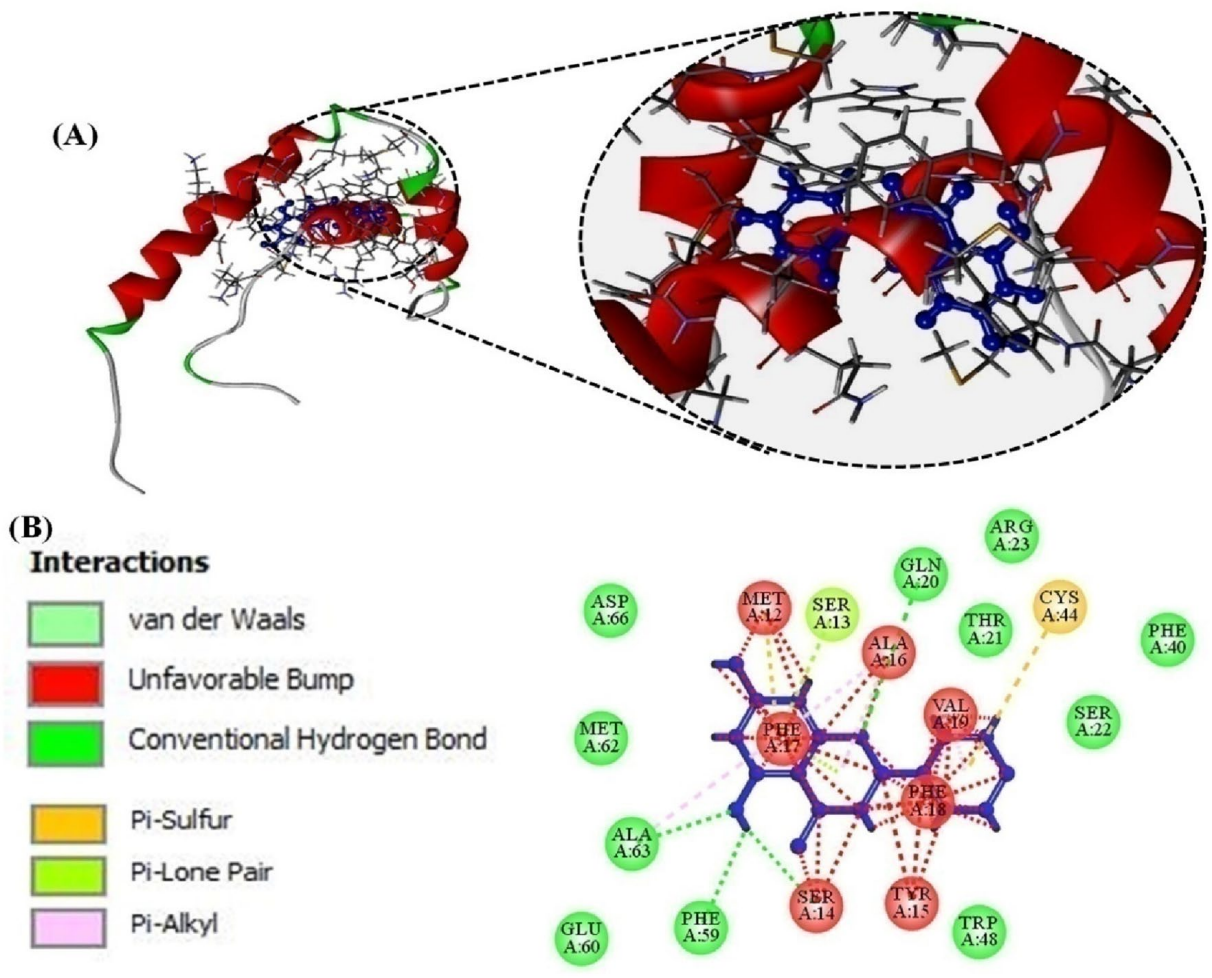
**A** Ball and socket cartoon model (blue) of the ligand (chrysin) interacting with the protein (HMGB1), **B** 2-D representation of various types of interactions (showed in color codes) of the ligand (chrysin) in ball and socket model (blue) with specific amino acid residues of the protein

Compound chrysin was docked with HMGB1 protein. The compound showed Gibbs free energy or binding free energy—5.7 kcal/mol that reflects a binding affinity of 14 × 10^3^ per mole (Table 1). Figure 8A provides a cartoon model of HMGB1 (red-green) as ball and stick pattern, and chrysin (blue) interacting with various amino acid residues of the receptor. Figure 8B provides 2D-plot representation of ligand (chrysin) interaction with amino acids including MET12, ASP 66, MET 62, ALA63, PHE59, GLU60, HIS33, GLN20, ARG23, CYS44,VAL19, PHE17, PHE18, SER14, SER22, PHE 40, TRP48, ALA16 and SER13, of the protein, via various types of interactions (conventional H-bonding, van der Waals forces, Pi-Pi T-shaped, Alkyl, Pi-alkyl, unfavourable acceptor-acceptor, unfavourable donor-donor bonds etc.). Table 1 provides the residues of the protein taking part in interactions with the ligands, shows the types of interactions, and bond distances in between the protein and the drug.

### Computational approaches for assessing physicochemical properties, drug-likeness and ADMET properties

We measured the drug potency and toxicity of tested compounds by employing admetSAR and SwissADME online web servers. The results of SwissADME and AdmetSAR web servers illustrates that the molecular weight (MW) of compounds lying within the range of 150-500 g/mol, plays a vital role in drug discovery [40]. Unquestionably, this property can affect several molecular activities like blood brain barrier permeation, absorption and relationship with on-and off-targets [41]. A smaller MW molecule would increase the rate of absorption and therefore most of the drugs are attempted to be maintained at the smallest possible MW. LogP_o/w_ (lipophilicity) is a common term used to evaluate lipophilicity and the partition coefficient between n-octanol and water, and its value must not exceed 5 (between range − 0.7 and 5.0). LogP_o/w_ is responsible for evaluating various ADMET properties including potency. For example, metabolism and solubility are compromised at higher lipophilicity values. The drug permeability is low when the LogP_o/w_ value is low [42]. Topological polar surface area (TPSA) value (range 20-150), is another parameter which suggests the prediction of transport properties or permeability of a candidate molecule in blood brain barrier (BBB), and the gastrointestinal tract. The drug permeability and oral bioavailability are significantly associated with hydrogen bonding (the number of oxygen and nitrogen atoms in a molecule) [40], 43. When passive diffusion makes way into CNS, the TPSA should be less than 80 Å^2^ [44]. The numbers of hydrogen bond acceptors (HBAs) and hydrogen bonds donors (HBDs) must be within 10 and 5 respectively. HBAs and HBDs are also essential parameters associated with polarity and permeability of candidate drugs [45]. By investigating variations in physicochemical properties of promoted oral drugs over time [46], it was demonstrated that MW and HBAs have considerably augmented, however HBDs and lipophilicity displayed comparatively little fluctuations. These findings propose that the overall sum of HBDs is more important than the count of HBAs [40] for drug designing and formulation, and can be associated with increasing bioavailability factor and membrane permeability [47]. Undoubtedly, for both these parameters, it was revealed that molecules holding higher count of HBAs with little HBDs have promising profile [48]. This is consistent with earlier studies which reveal that HBDs are usually the “foe of drug chemists”. Higher count of HBDs may be the reason of deprived permeability, absorption and bioavailability [49].

In the present study, MW of chrysin is within the acceptable range of 254.24 g/mol, LogP is also within the satisfactory range of 2.27, the TPSA score is 70.67 Å^2^ which clearly indicates better permeability into tissue. The number of HBA and HBD are 4 and 2 respectively for chrysin. For the positive control lamivudine, MW is 229.26 g/mol, LogP (lipophilicity) is within desirable range 1.04, TPSA is 115.67 Å^2^, the number of HBA and HBD are 4 and 2 respectively (Table 3). Natural compound chrysin and nucleoside analogue lamivudine were found to follow Lipinski’s rule of 5 (RO5) (Table 2). The RO5 is a mnemonic tool meant for a quick evaluation of molecules to assess their drug-likeness features during drug optimization and discovery. The rule claims that a molecule is expected to show low penetration when a couple of following physicochemical parameters are violated: MW> 500, LogP> 5, HBD> 5, and HBA> 10. The selected compound chrysin and the positive control followed the RO5 with zero violations as mentioned above. Table 2 demonstrates the ADMET profiles of chrysin and lamivudine. Log S implies the solubility of ligands that ideally varies between − 6.5 and 0.5. Chrysin and lamivudine are showing Log S values within the satisfactory range of − 4.19 and − 1.02, respectively. Both CaCo-2 (colorectal carcinoma) and Blood-Brain Barrier (BBB) permeability could be used to evaluate membrane permeability. BBB value represents its well permeation and delivery into the central nervous system (CNS). Blood−brain barrier is the boundary that separates the brain cells from the bloodstream, and serves a crucial role in safeguarding the CNS. It is largely made by the endothelium of brain. It impedes giant molecules by 100% and tiny molecules by 98% from piercing into the CNS, and allows the transport of only water- and lipid-soluble molecules, including selective transport molecules to pass through. The BBB satisfactory values for a suitable drug molecules range between − 3.0 and 1.2 [50]. Lamivudine violated and chrysin followed the BBB value under this ideal range. Both human intestinal absorption (HIA) and gastrointestinal (GI) absorption are positive for both chrysin and lamivudine indicating the absorption of the drugs are good, and are devoid of side effects. Drug metabolism through CYP isoenzymes plays a predominant role in drug interactions which could lead to drug toxicities and diminishing the pharmacological functions. Different cytochrome P450 inhibitors and substrates were studied, and the data are shown in (Table 2). Substrate of P-Glycoprotein (P-gp) serves as a drug eliminating pump which requires energy in the xenobiotic metabolism. Increased P-gp expression is observed in a variety of normal tissues, for example, liver, kidneys (renal tubules), colon, pancreas and adrenal cortex. These reports imply that P-gp may have a secretory physiological function [51]. The P-gp substrate was studied in the present study as shown in (Table 2). In case of toxicity, the tested ligand as well as positive control is labelled as noncarcinogenic and non-toxic**.** From the Ames mutagenicity test, none of ligands are mutagenic and exhibited a negative response to the Ames test for mutagenicity. The investigation of ADMET profiles of chrysin and lamivudine is important for their clinical use and commercial success as potential CHB drugs. The ADMET results proposed that chrysin can be used as an inhibitor molecule for treatment of chronic HBV (Table 2). Hence, admetSAR and SwissADME servers were important for the determination of molecular, physiochemical and pharmacokinetics properties of drugs.

**Table 2:** ADMET profile of lamivudine and chrysin estimated from SwissADME and admetSAR

Properties	Lamivudine	Chrysin
Absorption	BBB^−^	BBB^+^
Blood–Brain barrier (BBB)		
GI–Absorption	High	High
Caco-2 permeability	Caco-2^−^	Caco-2^+^
Human intestinal absorption	HIA^+^	HIA^+^
Metabolism	No	Yes
CYP1A2 inhibitor		
CYP2C19 inhibitor	No	No
CYP2C9 inhibitor	No	No
CYP2D6 inhibitor	No	Yes
CYP3A4 inhibitor	No	Yes
P-gp substrate(P-glycoprotein)	No	No
Toxicity	Non-toxic	Non-toxic
Ames toxicity		
Carcinogenicity	Non-carcinogenic	Non-carcinogenic
Rule-of-five (RO5)	Not violated	Not violated
Lipinski		
Ghose	Violated	Not violated
Veber	Not violated	Not violated
Egan	Not violated	Not violated
Muegge	Not violated	Not violated

## Discussion

The advent of nucleos(t)ide analogues resistance mutations in the viral polymerase represents a major drawback of the currently available antiviral drugs for treating patients with chronic hepatitis B [52]. Because of this, there is a critical need to develop novel antiviral treatments. Natural flavonoids, alkaloids, saponins, polyphenols, terpenoids and lignans have been documented for potential anti-HBV activities [53, 54]**,** either by means of directly impeding viral replication or regulating host immune‐response [55]. Our study for the first time shows anti-HBV potential of chrysin by in vitro studies. The results are further supported by molecular docking and ADMET analysis. This study is also the first evidence of possible mechanism of action of anti-HBV activity of chrysin. MTT assay (Fig. 2) and subsequent in vitro experiments (Figs. 3, 4, 5, 6) revealed that chrysin had anti-HBV potential at safe doses. Anti-HBV potential of chrysin is correlated with other flavonoids containing broad spectrum of pharmacological properties. Among them one is epigallocatechin-3-gallate (EGCG), a compound derived from green tea. At a concentration of 50 μmol/L, EGCG decreases HBV-host cell entry by > 80% [56]. Further investigations revealed that in a dose and time dependent manner, EGCG had significantly affected HBV life cycle by reducing HBsAg, HBeAg and extracellular HBV DNA in HepG2.2.15 [57, 58]. Wogonin, another flavonoid from *Scutellaria radix*, efficiently decreases secretion of HBsAg, HBeAg, HBV DNA and viral replication in HepG2.2.15. In humanized HBV-transgenic mice, wogonin substantially decreases HBsAg secretion [59]. In line with these reports, our results show that chrysin decreases HBsAg and HBeAg secretion, extracellular HBV DNA and intracellular cccDNA, in a dose dependent manner, after 72 h post treatment.

Glycyrrhizin has been used for a long time in Japan to treat chronic hepatitis patients. This compound is the first to be reported to target HMGB1 in chronic hepatitis. Our study compound chrysin is very similar to glycyrrhizin, because both are plant derivatives, and exhibit broad-spectrum pharmacological attributes, like antioxidant, anti-cancer, anti-inflammatory and hepatoprotective activities [60–62]. In line with these reports, our in silico study, where chrysin and lamivudine (a positive control) are compared for their binding affinities for HMGB1, also demonstrate significant chrysin-HMGB1 binding.

HMGB1 is a well reported cytokine actively involved in increasing the level of proinflammatory molecules such as IL-17 and NF-κB etc., in context of HBV pathogenesis [63]. Several antivirals natural compounds like glycyrrhizin, quercetin and EGCG, that are similar natural plant derivatives as chrysin, are reported to interact directly with HMGB1, and reduce chronic inflammation [64]. Glycyrrhizin is further reported to decrease HBV related chronic liver inflammation via interacting with HMGB1 [60–62]. Therefore HMGB1 is an important target for docking studies as has already been mentioned in several reports.

To elucidate and assess the mechanism of action of chrysin as an anti-HBV drug, and the molecular interaction paradigm, we further investigated HMGB1 inhibition by chrysin. This was performed by correlating chrysin with similar natural compounds-quercetin and EGCG. Quercetin is a flavonoid found in fruits and vegetables. In studies involving macrophage cultures, quercetin is reported to inhibit the release of HMGB1 and its cytokine activities, and restricted mitogen-activated protein kinase (MAPK) activation [65]**.**

Another plant derivative, epigallocatechin-3-gallate (EGCG), reduces fatal endotoxemia during systemic inflammation. EGCG, present in *Camellia sinensis* (component of green tea)*,* protects mice from endotoxemia and fatal sepsis. This is done by reducing HMGB1 formation and release in macrophage cultures, and preventing the clumping of exogenous HMGB1 on macrophage cell surface. The clumping is a key player for HMGB1 induced inflammatory responses [66]. In chronic liver inflammation, HMGB1 plays an important role by enhancing the concentration of proinflammatory cytokines, like IL-17. This cytokine surge encourages infiltration of neutrophils and liver inflammatory response [67]. Up regulation of HMGB1is linked with severity of chronic HBV disease [68]. HMGB1 increases pro-IL-1β levels in macrophages by activating Toll-like receptors (TLR4) signalling, nuclear factor kappa B (NF-κB) and p38 mitogen-activated protein kinase (MAPK) pathways [69]. NF-κB (immune response regulator), p38 and MAPK are associated with inflammation. The receptor for advanced glycation end products (RAGE), a transmembrane protein of immunoglobulin superfamily, that stimulates both innate and adaptive immunity, functions as molecular pattern recognition receptor**.** The HMGB1 amino acids 150–183 are responsible for RAGE firm binding. The receptor for RAGE interacts with HMGB1 and increases the inflammation rate [70]. It is reported that RAGE activates p38, NF-κB, and MAPK, allowing release of proinflammatory cytokines [71, 72]. Amino acids 89–108 of HMGB1 are responsible for its binding with TLR4 [73]. Therefore, impeding HMGB-1/TLR4 signaling pathway at plasma membrane is effective therapeutic strategy for alleviating chronic HBV infection and reducing liver inflammation. HMGB1 plays predominant role in etiology of liver failure in chronic HBV individuals by impeding immunological activity of regulatory T cells and by down-regulating Foxp3 expression [74].

Docking of lamivudine exhibited that it had significantly bound with HMGB1 active site via generating a rigid lamivudine-HMGB1 complex with an estimated Gibb’s free energy of 4.3 kcal/mol and binding affinity K_b_ 1.0 × 10^3^ per mole (Table 1). Docking studies (Fig. 7) further proved that lamivudine interacted with HMGB1 by classical and non-classical interactions. Lamivudine interacts with HMGB1 via developing bonds with residues GLN20, MET12, SER13, SER14, PHE17, PHE59, ALA16, VAL19, TYR15, and TRP48.

In order to understand intermolecular interactions and strength of ligand–protein interactions, binding affinities (*K*_b_) of each ligand (chrysin and lamivudine) with the protein (HMGB1) were calculated. *K*_b_ is responsible for biological processes, structural biology, and structure–function correlations [75]. Intermolecular weak contacts, such as electrostatic interactions, van der Waals forces, hydrogen bonding, and hydrophobic interactions influence binding affinity. Details of amino acids of the protein that interacted with the drugs (chrysin and lamivudine) are provided in Table 2. The binding sites for each drug were different on the protein and also the different types of interactions took place.

Better understanding of docked protein HMGB1 and inhibitory potential of candidate antiviral molecules could be important breakthrough in exploring treatment options for chronic HBV disease. Strikingly, cellular DNA binding and bending activities are regulated by HMGB1 (serves as chaperone of DNA) [76, 77]. HMGB1 bends DNA and modifies its conformation by unwinding, looping or compacting (Javaherian et al*.* [77]). Accordingly, we hypothesize that upon HMGB1 binding, HBV DNA may alter its conformation. This change might favor recruitment of regulatory proteins, and inducing HBV replication. In case of viral hepatitis, we hypothesize that chrysin may reduce liver inflammation via HMGB1-TLR4 signaling pathway, in a manner as reported for glycyrrhizin [78]. We postulate that trapping of HMGB1 by chrysin contributes in viral infections by decreasing the level of proinflammatory cytokines induced by HMGB1. We further speculate that chrysin can down regulate expression of inflammatory cytokines and related proteins involved in chronic HBV infection.

When subjected to in silico investigations, chrysin made stable interaction with HMGB1 via MET12, ASP 66, MET 62, ALA63, PHE59, GLU60, HIS33, GLN20, ARG23, CYS44, VAL19, PHE17, PHE18, SER14, SER22, PHE 40, TRP48, ALA16 and SER13 with an approximate binding free energy of − 5.0 kcal/mol and binding affinity (K_b_) of 14 × 10^3^ per mole (Table 1) (Fig. 8).

Various research groups demonstrated relationship between physicochemical properties, ADMET profiles and effectiveness of tiny molecules. Drug candidates must be amply permeable and soluble to reach their targets for action. Physicochemical properties calculated for chrysin are MW, logS, LogP, TPSA, HBD, HBA, number of rotatable bonds and molar refractive index (Table 3). As per Lipinski’s rule of five, ligands with less absorption or penetration have hydrogen bond acceptors (HBA) > 10, hydrogen bond donor(HBD) > 5, MW > 500 Da and estimated lipophilicity (LogP) > 5 [79]. It is reported that ligands with rotatable bonds (nrotb) ≤ 10 and total polar surface area (TPSA) of ≤ 140 Å have high bioavailability [80]. In the present study, LogP of chrysin is 2.27, and HBA and HBD are 4 and 2, respectively, which means higher chances of oral bioavailability, absorption and penetration (Table 3).

**Table 3:** Physicochemical properties of chrysin and lamivudine calculated by SwissADME

Properties	Lamivudine	Chrysin
CAS (chemical abstract service) number	134678-17-4	480-40-0
Molecular formula	C_8_H_11_N_3_O_3_S	C_15_H_10_O_4_
Molecular weight (MW)	229.26	254.24
Log S (solubility)	− 0.84	− 4.19
LogP (lipophilicity octanol/water)	1.04	2.27
Topological polar surface area (TPSA)	115.67 Å^2^	70.67 Å^2^
Number of H-bond acceptors (HBAs)	4	4
Number of H-bond donors (HBDs)	2	2
Molar refractive index	56.31	71.97
Number of rotatable bonds (nrotb)	2	1

Compounds with MW of ≤ 500 Da are rapidly transported, diffused and absorbed as compared to higher MW candidate compounds [81]**.** In the present study, chrysin and lamivudine fell within the desirable range of MW and therefore easily crosses BBB (Table 3). ADMET parameters of the ligands were demonstrated by using  SwissADME and admetSAR. Parameters such as penetration of BBB, HIA, Caco-2 cell permeability, Ames test and carcinogenicity were measured. Results show that chrysin and lamivudine can be absorbed by the human intestine, penetrates Caco-2 cell (Table 2) and crosses BBB.

Lamivudine was demonstrated to be a potential substrate for P glycoprotein (P-gp), a biomolecule that effluxes drugs and other compounds for subsequent metabolism and clearance [82]. Human microsomal cytochrome P450s (CYP) is responsible for the catalysis of a diverse xenobiotics and pharmaceuticals [83]. Results of Ames test show that chrysin and lamivudine do not display severe toxicity, mutagenicity and carcinogenicity (Table 2).

## Conclusions

To conclude, chrysin exhibits the antiviral potential against HBV by lowering HBeAg, HBsAg expressions, extracellular HBV DNA and intracellular cccDNA, in a dose dependent manner. The in vitro investigations were further validated by computational docking analysis (Table 1, Figs. 7A and 8A), which shows that chrysin and lamivudine strongly interact with the active site residues of HMGB1. The interaction of chrysin with HMGB1 might be responsible for its antiviral activities. Analysis of ADMET parameters (Table 2) and physicochemical properties (Table 3) shows that chrysin and lamivudine are suitable for humans without any carcinogenic, mutagenic, and toxic effects. To use chrysin as a treatment of chronic HBV disease, further endorsement and optimization of results by in vivo studies in animal models are needed.

## Data Availability

The datasets used and/or analyzed during the current study are available from the corresponding author on reasonable request.

## Abbreviations

ADMET: Absorption, distribution, metabolism, excretion and toxicity
BBB: Blood–brain barrier
CNS: Central nervous system
CccDNA: Covalently closed circular DNA
DMSO: Dimethylsulphoxide
ELISA: Enzyme linked immunosorbent assay
HBV: Hepatitis B virus
HCC: Hepatocellular carcinoma
HBAs: Hydrogen bond acceptors
HBDs: Hydrogen bond donors
HIA: Human intestinal absorption
HMGB1: High mobility group box 1 protein
MTT: 3-(4, 5-Dimethyl-2-thiazolyl)-2, 5-diphenyl-2-H-tetrazolium bromide
NAs: Nucleoside analogues
Ro5: Rule of five
SD: Standard deviation
YMDD: Tyrosine-methionine-aspartate-aspartate
